# OpenNotes After 7 Years: Patient Experiences With Ongoing Access to Their Clinicians’ Outpatient Visit Notes

**DOI:** 10.2196/13876

**Published:** 2019-05-06

**Authors:** Jan Walker, Suzanne Leveille, Sigall Bell, Hannah Chimowitz, Zhiyong Dong, Joann G Elmore, Leonor Fernandez, Alan Fossa, Macda Gerard, Patricia Fitzgerald, Kendall Harcourt, Sara Jackson, Thomas H Payne, Jocelyn Perez, Hannah Shucard, Rebecca Stametz, Catherine DesRoches, Tom Delbanco

**Affiliations:** 1 Division of General Medicine Beth Israel Deaconess Medical Center Boston, MA United States; 2 Harvard Medical School Boston, MA United States; 3 College of Nursing and Health Sciences University of Massachusetts Boston, MA United States; 4 David Geffen School of Medicine University of California Los Angeles Los Angeles, CA United States; 5 Division of General Internal Medicine School of Medicine University of Washington Seattle, WA United States; 6 Department of Medicine Medicine Information Technology Services University of Washington Seattle, WA United States; 7 Steele Institute for Health Innovation Geisinger Danville, PA United States

**Keywords:** patient portal, physician-patient relations, electronic health record, health care survey, patient participation

## Abstract

**Background:**

Following a 2010-2011 pilot intervention in which a limited sample of primary care doctors offered their patients secure Web-based portal access to their office visit notes, the participating sites expanded OpenNotes to nearly all clinicians in primary care, medical, and surgical specialty practices.

**Objective:**

The aim of this study was to examine the ongoing experiences and perceptions of patients who read ambulatory visit notes written by a broad range of doctors, nurses, and other clinicians.

**Methods:**

A total of 3 large US health systems in Boston, Seattle, and rural Pennsylvania conducted a Web-based survey of adult patients who used portal accounts and had at least 1 visit note available in a recent 12-month period. The main outcome measures included patient-reported behaviors and their perceptions concerning benefits versus risks.

**Results:**

Among 136,815 patients who received invitations, 21.68% (29,656/136,815) responded. Of the 28,782 patient respondents, 62.82% (18,081/28,782) were female, 72.90% (20,982/28,782) were aged 45 years or older, 76.94% (22,146/28,782) were white, and 14.30% (4115/28,782) reported fair or poor health. Among the 22,947 who reported reading 1 or more notes, 3 out of 4 reported reading them for 1 year or longer, half reported reading at least 4 notes, and 37.74% (8588/22,753) shared a note with someone else. Patients rated note reading as very important for helping take care of their health (16,354/22,520, 72.62%), feeling in control of their care (15,726/22,515, 69.85%), and remembering the plan of care (14,821/22,516, 65.82%). Few were very confused (737/22,304, 3.3%) or more worried (1078/22,303, 4.83%) after reading notes. About a third reported being encouraged by their clinicians to read notes and a third told their clinicians they had read them. Less educated, nonwhite, older, and Hispanic patients, and individuals who usually did not speak English at home, were those most likely to report major benefits from note reading. Nearly all respondents (22,593/22,947, 98.46%) thought Web-based access to visit notes a good idea, and 62.38% (13,427/21,525) rated this practice as very important for choosing a future provider.

**Conclusions:**

In this first large-scale survey of patient experiences with a broad range of clinicians working in practices in which shared notes are well established, patients find note reading very important for their health management and share their notes frequently with others. Patients are rarely troubled by what they read, and those traditionally underserved in the United States report particular benefit. However, fewer than half of clinicians and patients actively address their shared notes during visits. As the practice continues to spread rapidly in the United States and internationally, our findings indicate that OpenNotes brings benefits to patients that largely outweigh the risks.

## Introduction

### Secure Patient Portals

Patients who engage and participate actively in their health care appear to achieve better health outcomes and incur lower health care costs [1]. In part, to stimulate such engagement, the US federal government passed a legislation in 2009 incentivizing doctors and health systems to adopt new technologies offering patients electronic access to their health data via secure electronic patient portals [2]. Patients were invited to review their test results and problem and medication lists and, in many cases, to send secure messages to their clinicians. However, very few offered patients access to the notes written by their clinicians during or following face-to-face encounters.

### The OpenNotes Initiative

In 2010, the Robert Wood Johnson Foundation funded the OpenNotes initiative, designed initially to examine the feasibility and effects of having primary care physicians (PCPs) share their notes routinely with patients. For 12 months, PCPs invited patients registered on portals to read these notes and 105 doctors and 5500 of their patients subsequently completed surveys. Representing 3 geographically dispersed and very different health care settings, the respondents were highly positive, with patients reporting a wide range of clinically important benefits and doctors noting little impact on their workflow [3]. Subsequent studies extending beyond primary care to medication adherence, inpatient care, oncology, mental illness, and other specialties suggested similar benefits, and in recent years, the practice of inviting patients to review their clinicians’ notes has spread [4-13]. Presently, US clinicians offer more than 38 million patients electronic access to their notes through patient portals, policy makers are considering mandating such practice, and fully transparent records are spreading in several other nations [14-16]. Many prominent American institutions now offer open notes in the vast majority of their ambulatory practices, including medical and surgical offices and those focusing on mental illness. However, at this point, we know little about patient experiences with open notes over time throughout the broad spectrum of ambulatory care.

Following their initial limited experiment in primary care, the 3 institutions that originally piloted OpenNotes adopted the practice throughout, and many of their patients have grown accustomed to reading notes following visits to nearly all clinicians in their associated primary care, medical, surgical, and mental health practices. On the basis of the reports and observations over the past few years, we developed 4 primary hypotheses: (1) over time, patients would continue to report important benefits from reading visit notes; (2) patients would often share or discuss notes with others; (3) those traditionally at risk for experiencing substandard care would report the greatest benefit; and (4) patients and clinicians would communicate about these notes actively during visits. We report findings from a large survey of patients conducted in the institutions that participated in the pilot OpenNotes inquiry.

## Methods

### Setting

We conducted a Web-based survey of patients who had been seen in hospital offices and community practices at 3 health systems: Beth Israel Deaconess Medical Center (BIDMC), an urban academic health system in and around Boston; Geisinger, a large rural integrated health system in Pennsylvania; and University of Washington Medicine (UW) in Seattle, which includes both private and community-funded safety net practices affiliated with the University of Washington. All 3 systems participated in the original 2010-2011 OpenNotes pilot involving PCPs [3] and, by 2014, all 3 had expanded open notes to virtually all outpatient offices and clinicians, thereby providing access to visit notes in specialty as well as primary care settings. Open notes became the standard for virtually all types of outpatient clinicians who sign notes in the patient’s medical record, including doctors, physical, occupational, speech, and other types of therapists, dieticians, nurses, nurse practitioners, and physician assistants. At the same time, the systems developed policies allowing some individual clinicians to opt out of participation and enabled participating clinicians to manually block the release of individual notes to the patient portal [17]. BIDMC and UW sent automated email messages informing patients when new notes were made available, but Geisinger did not [18]. At the time of the survey, somewhat fewer than half of all ambulatory patients were registered for the patient portal at each site (personal communications from Rebecca Stametz and Thomas Payne, July 24, 2018, and Amy Goldman, July 25, 2018).

### Participants

The survey included patients seen in primary care and specialty offices: at the hospital and 6 affiliated sites at BIDMC, at 3 hospitals and 9 freestanding offices at UW, and at 7 hospitals and 53 outlying practices at Geisinger. Eligible patients were aged 18 years or older, had logged into the portal at least once in the previous 12 months, and had at least 1 ambulatory visit note available in the previous 12 months. We excluded patients who had been invited to participate in focus groups or other surveys related to OpenNotes within the preceding 12 months. Using portal tracking data, we identified patients who had, and had not, accessed available visit notes in the previous 12 months and described them as readers and nonreaders, respectively. We did not exclude nonreaders because we wanted to gain some understanding of why they had not read the notes. BIDMC and UW included all eligible readers and a random sample of eligible nonreaders in the survey sample. For administrative reasons, Geisinger drew random samples from both groups, resulting in smaller samples than the other 2 sites. Across the 3 sites, 109,904 readers and 27,959 nonreaders were sent invitations for the survey. The Institutional Review Boards at BIDMC, Geisinger, and UW approved the survey and study protocol at their respective sites.

### Constructing the Questionnaire

This survey draws heavily on questions used in the original demonstration project in primary care; its development has been previously described [19]. We updated the questionnaire based on comments from outside reviewers, focus groups with diverse patients from a community health center, assessment of the distribution of responses in the original study, and an evaluation of psychometric properties of different versions of some of the items, including those related to benefits and risks of reading notes (manuscript under review). Except for a few site-specific modifications, the questionnaire was the same for all patients, and with the exception of skip patterns, free text, and demographic questions, all items required a response. Both the original and updated versions are available on request from the authors.

### Conducting the Survey

We surveyed patients between June and October 2017, using a Web-based survey platform, Survey Gizmo. Patients were sent invitations by email either to their portal accounts or to the personal email address associated with the accounts. Each patient’s invitation contained his or her study identification embedded in a unique link to the survey, and each study identification could be used only once. Following the original invitation, patients received 2 reminders 1 week apart if they had not completed the survey. Knowing that patients sometimes confuse notes with other parts of the medical record, or with the portal itself, we described clinical notes in both the survey invitation and in a survey question and we also showed a screenshot of the location of visit notes on their institutions’ portals to increase the likelihood that patients would report on visit notes. Knowing also that invitations might be opened by care partners rather than patients, each respondent had an option to complete the survey as a patient or as a care partner, and care partners were automatically linked to a different questionnaire. We offered respondents an incentive for completing the survey: a raffle of 50 prizes of US $25 or US $50 at each site.

### Statistical Analysis

To maximize the chances that we were including responses about clinical notes rather than another part of the record, as a final step, we excluded responses from patients whose self-report of note reading in the past 12 months did not match portal data; for example, patients reported they had read notes, but the portal tracking data showed they had not. We also excluded respondents who reported reading notes for a week or less, or did not answer the question about length of time reading notes, since our objective was to assess patients’ experiences over the prior 12 months. Except as noted in the tables, all questions included in this analysis had < 4% missing responses, and denominators include all nonmissing responses to each item. Items related to medication management are addressed in another paper [20].

Most items addressing potential benefits and risks asked for ratings on an 11-point scale, ranging from 0 (not at all important, confusing, or concerned) to 10 (extremely important, confusing, or concerned). Responses to these items were collapsed into 4 categories a priori for analysis: 0-1, 2-4, 5-7, and 8-10, and we reported the 8-10 category as *very important, confused, or concerned*. Four-level agreement responses were dichotomized as agree or somewhat agree, and disagree or somewhat disagree.

Using percentages and chi-square tests, we compared respondents with nonrespondents using administrative data available at each institution: age and sex at all 3 sites, Hispanic ethnicity at Geisinger and UW, and insurance type at BIDMC and Geisinger.

We used descriptive statistics to examine respondents’ sociodemographic and health characteristics and experiences with note reading, both overall and stratified by study site. We used the chi-square test for independence (degrees of freedom: row-1 × column-1) to test for differences according to demographic characteristics in patients’ experiences in note reading. We performed a multivariable analysis using log Poisson regression models to estimate overall relative risks and 95% CIs for reporting benefits as very important according to patient characteristics, adjusted for other sociodemographic and health characteristics and number of notes read. Owing to the large sample size, we expected even slight differences between groups to be statistically significant. Therefore, we interpreted intercategory differences in proportions or relative risks of 10% or more as meaningful differences. All analyses were completed using SAS software version 9.4 (SAS Institute Inc).

## Results

### Participants

**Figure 1:**
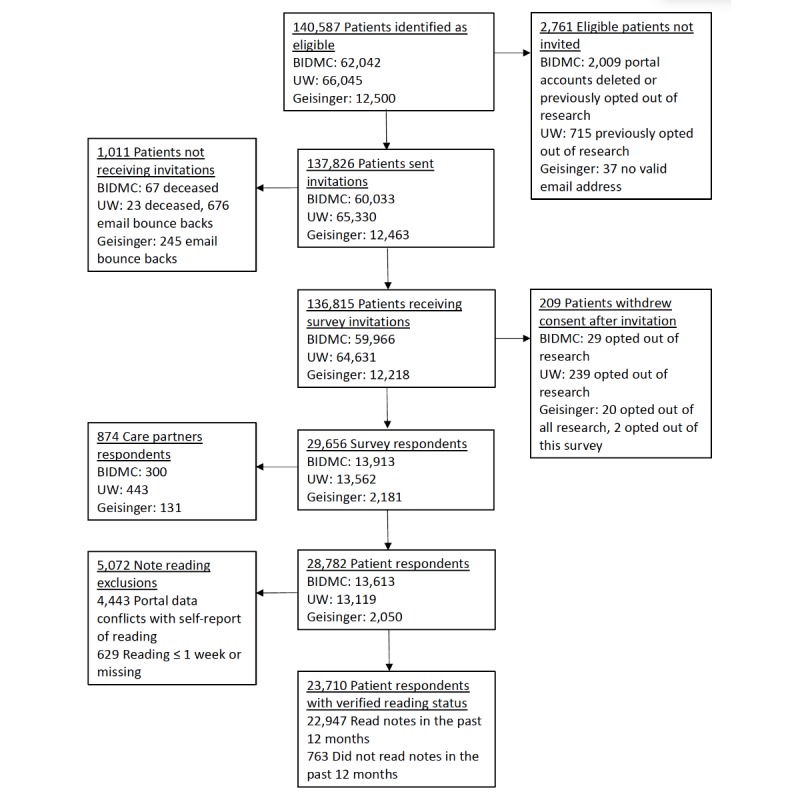
Study flow diagram. BIDMC: Beth Israel Deaconess Medical Center; UW: University of Washington Medicine.

Of 136,815 patients who received survey invitations, 21.68% (29,656/136,815) responded; 28,782 were patients, and 874 were care partners (Figure 1). Compared with nonrespondents, respondents were older at all 3 sites. At BIDMC and UW, white patients were more likely to respond than nonwhite patients. No differences were noted between responders and nonresponders regarding sex at any of the 3 sites, regarding Hispanic ethnicity at UW and Geisinger, and regarding insurance type at BIDMC and Geisinger (data not shown).

Of the 28,782 patient respondents, 62.82% (18,081/28,782) were female; 72.90% (20,982/28,782) were aged 45 years or older; 76.94% (22,146/28,782) were white; 53.06% (15,271/28,782) were employed; and 14.30% (4115/28,782) reported fair or poor general health. Overall, 64.92% (18,685/28,782) had completed college, but the sites differed substantially: the proportion of those with a high school education or less was 4.8% (655/13,613) at BIDMC and 5.4% (708/13,119) at UW, but 25.9% (530/2050) at Geisinger.

### Accessing and Reading Notes

After note reading exclusions, 23,710 responses were included in the analysis: 22,947 note readers and 763 nonreaders. Among note readers, three-quarters reported reading notes for a year or more and half reported reading 4 or more notes. In general, they reported locating notes easily on the portals and considered email notifications about new notes useful. About one-third of readers at BIDMC (4065/11,899, 34.16%) and UW (2998/9719, 30.85%), but only 20.1% (237/1178) at Geisinger, reported mentioning to their clinicians that they had read a note, and about the same proportion (7324/22,798, 32.13%) said they were encouraged by their clinicians to read notes. Among those who were encouraged, 60% read 4 or more notes compared with 46% of those who were not encouraged to read notes. In total, 37.74% (8588/22,753) reported sharing or discussing a note with a family member or someone else. Overall, 98.46% (22,593/22,947) of readers thought making notes available to patients a good idea, and 62.38% (13,427/21,525) said access to visit notes would be very important in choosing a future provider.

### Benefits and Risks of Note Reading

**Figure 2:**
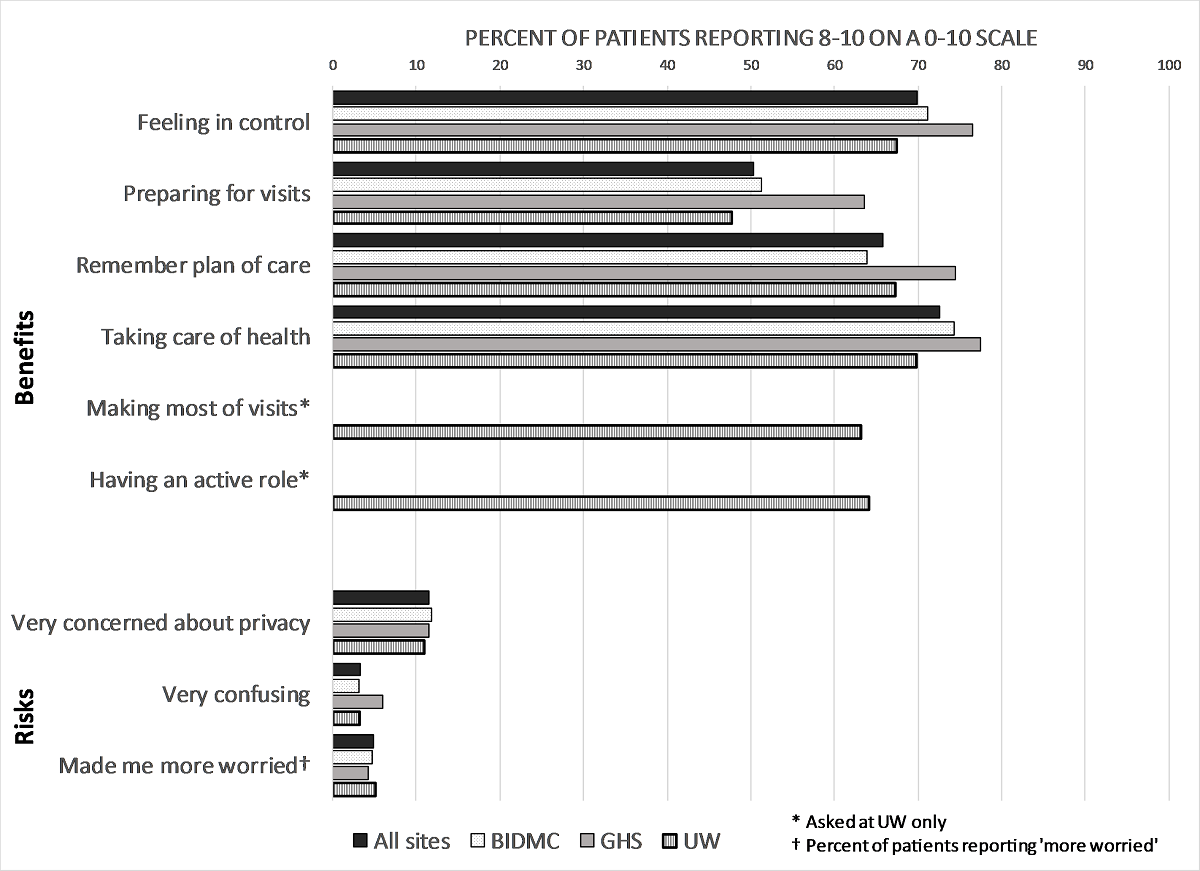
Benefits and risks of reading notes. BIDMC: Beth Israel Deaconess Medical Center; UW: University of Washington Medicine.

As shown in Figure 2, between 50.43% (11,346/22,514) and 72.62% (16,354/22,520) of patients in all sites combined rated note reading as very important for helping them achieve 6 different benefits. In contrast, 3.3% (737/22,304) said they were very confused by their notes, 4.83% (1078/22,303) reported being more worried after reading notes, and 11.46% (2529/22,067) reported being very concerned about privacy. The positive perceptions of benefits were evident across the 3 sites, with patients in rural Pennsylvania reporting the highest ratings of importance of note reading.

We found meaningful differences (≥ 10%) in patients’ perceptions of the benefits of reading notes according to sociodemographic characteristics (Table 1). Black patients were more likely than white patients to rate note reading as very important for 5 of the 6 benefits and patients aged 45 years or older rated it very important for 4 of the benefits compared with those aged 18 to 24 years. Those who usually spoke a language other than English at home were more likely than English speakers to use notes to make the most of visits, remember the plan of care, and prepare for visits. Patients with the fewest years of education and Hispanic patients were more likely than others to cite note reading as very important for remembering the care plan and preparing for visits. Patients who read a greater number of notes were more likely to cite reading notes as very important for all 6 benefits.

Few patients reported being very confused or more worried from reading notes, with only minor differences according to sociodemographic characteristics (Table 2). Though differences did not reach the 10% threshold, greater proportions of black patients, Asians, and other minorities reported being very concerned about privacy related to open notes, 16.2% (174/1076) to 19.2% (104/543), compared with 10.15% (1828/18,012) of white patients.

After adjusting for other factors, education level continued to be inversely associated with patient ratings of the importance of note reading for all 6 benefits (Table 3). Similarly, after adjusting for other characteristics, black patients were more likely than white patients to rate note reading as very important for achieving benefits. Older patients—especially those aged > 45 years—were more likely to rate notes as important for 4 of the benefits. Hispanic patients and those who spoke a language other than English at home found notes very important for 3 benefits. Notably, the importance of note reading in preparing for office visits was the benefit most endorsed among more vulnerable demographic groups.

When we asked the 763 nonreaders about the main reason they had not looked at visit notes, about half selected, *I forgot or did not know my visit notes were available*. A total of 10.4% (79/761) indicated they did not know they had a right to look at notes, 8.8% (67/761) were too busy, 7.2% (55/761) did not think reading would be useful, and 6.3% (48/761) were not able to find the notes. Among the 12.0% (95/761) reporting another reason, the majority wrote that they had no need to read notes because they trusted their clinicians, had received printed copies, or had no health issues or recent visits. Only 9.1% (69/760) of nonreaders reported being encouraged by a clinician to read their notes. We found no material differences in demographic characteristics in the nonreaders compared with the readers (data not shown). Even though they had not read notes, 89.0% (679/763) of nonreaders agreed that making notes available to patients on the Web is a good idea.

**Table 1:** Proportion of patients identifying notes as *extremely important* in achieving benefits.

Demographics		Taking care of health^a^		Active role in care^a,b^		Feeling in control of care^a^		Making the most of visits^a,b^		Remembering the plan of care^a^		Preparing for visits^a^	
		n (%)	*P* value^c^	n (%)	*P* value^c^	n (%)	*P* value^c^	n (%)	*P* value^c^	n (%)	*P* value^c^	n (%)	*P* value^c^
**Age (years)**			**<.001**		**<.001**		**.007**		**<.001**		**.004**		**<.001**
	18-24	436 (58.80)		233 (53.60)		498 (67.10)		227 (52.30)		473 (63.80)		303 (40.80)	
	25-44	3358 (67.63)		1471 (62.38)		3452 (69.54)		1392 (59.06)		3263 (65.75)		2249 (45.32)	
	45-64	7073 (75.47)		2453 (66.07)		6652 (70.99)		2411 (64.86)		6285 (67.07)		4893 (52.22)	
	65+	5487 (73.74)		1977 (64.88)		5124 (68.88)		2003 (65.76)		4800 (64.52)		3901 (52.44)	
**Sex**			**.14**		**<.001**		**<.001**		**.06**		**<.001**		**.15**
	Female	10,376 (72.96)		3954 (65.99)		10,212 (71.82)		3829 (63.87)		9604 (67.54)		7217 (50.76)
	Male	5978 (72.04)		2180 (61.22)		5514 (66.46)		2204 (61.93)		5217 (62.89)		4129 (49.76)	
**Race**			**<.001**		**.01**		**<.001**		**<.001**		**<.001**		**<.001**
	White	13,012 (72.24)		4757 (64.03)		12,523 (69.53)		4653 (62.63)		11,726 (65.1)		8918 (49.51)
	Black	455 (83.80)		93 (73.00)		436 (80.30)		93 (73.00)		423 (77.90)		342 (63.00)	
	Asian	824 (76.60)		433 (67.30)		797 (74.10)		443 (68.90)		777 (72.20)		597 (55.50)	
	Other	535 (76.00)		236 (68.80)		522 (74.20)		233 (67.90)		511 (72.60)		424 (60.20)	
	Multiple races	578 (75.70)		322 (69.00)		560 (73.30)		317 (67.90)		535 (70.00)		420 (55.00)	
**Ethnicity**			**<.001**		**.31**		**<.001**		**.54**		**<.001**		**<.001**
	Hispanic or Latino	626 (80.50)		217 (67.40)		612 (78.70)		210 (65.20)		599 (77.00)		472 (60.70)
	Non-Hispanic	14,845 (72.69)		5641 (64.66)		14,282 (69.93)		5545 (63.56)		13,426 (65.74)		10,271 (50.29)	
**Education**			**<.001**		**<.001**		**<.001**		**<.001**		**<.001**		**<.001**
	Masters or doctoral degree	5713 (71.33)		1958 (62.12)		5410 (67.55)		1923 (61.01)		4873 (60.84)		3700 (46.20)
	4-year college degree or some graduate school	5210 (71.33)		2052 (64.33)		5101 (69.84)		2018 (63.26)		4824 (66.05)		3526 (48.27)	
	Some college or technical school	3591 (76.40)		1563 (68.07)		3450 (73.40)		1533 (66.77)		3413 (72.62)		2719 (57.85)	
	High school or less	1113 (79.73)		335 (68.90)		106 (76.50)		336 (69.10)		1053 (75.43)		902 (64.60)	
**Language**			**<.001**		**.03**		**<.001**		**.002**		**<.001**		**<.001**
	Other	372 (80.50)		142 (72.10)		361 (78.10)		146 (74.10)		357 (77.30)		315 (68.20)
	English	15,136 (72.84)		5728 (64.58)		14,567 (70.10)		5627 (63.44)		13,700 (65.93)		10,466 (50.36)	
**General health**			**.08**		**.19**		**.07**		**.97**		**<.001**		**<.001**
	Excellent, very good, or good	13,134 (72.76)		4853 (65.09)		12,717 (70.45)		4752 (63.73)		11,853 (65.66)		9050 (50.14)
	Fair or poor	2452 (74.21)		1047 (63.38)		2275 (68.86)		1052 (63.68)		2283 (69.10)		1778 (53.81)	
**Number of notes read**			**<.001**		**<.001**		**<.001**		**<.001**		**<.001**		**<.001**
	1	869 (54.80)		429 (51.40)		892 (56.20)		421 (50.40)		830 (52.30)		552 (34.80)
	2 or 3	5815 (66.86)		2244 (59.81)		5619 (64.64)		2176 (57.96)		5251 (60.40)		3780 (43.48)	
	4 or more	9098 (80.56)		3223 (71.34)		867 6 (76.83)		3202 (70.89)		8194 (72.55)		6615 (58.59)	
	Do not know or not sure	572 (60.70)		238 (53.00)		539 (57.20)		234 (52.20)		546 (57.90)		399 (42.30)	

^a^Answering 8 to 10 on a 0-10 scale.

^b^Only asked at University of Washington Medicine.

^c^*P* values from the chi-square test for independence between categorical measures.

**Table 2:** Proportions of patients reporting risks from reading notes.

Demographics		Notes were very confusing^a^		Very concerned about privacy^a^		More worried after reading notes^b^	
		n (%)	*P* value^c^	n (%)	*P* value^c^	n (%)	*P* value^c^
**Age (years)**			**<.001**		**<.001**		**<.001**
	18-24	8 (1.00)		48 (7.00)		46 (6.00)
	25-44	112 (2.30)		433 (9.00)		256 (5.20)	
	45-64	285 (3.10)		1284 (13.96)		471 (5.10)	
	65+	332 (4.50)		764 (10.40)		305 (4.10)	
**Sex**			**.23**		**<.001**		**<.001**
	Female	481 (3.40)		1488 (10.68)		721 (5.10)
	Male	256 (3.10)		1041 (12.81)		357 (4.40)	
**Race**			**.72**		**<.001**		**<.001**
	White	570 (3.20)		1828 (10.10)		807 (4.50)
	Black	17 (3.10)		104 (19.20)		32 (6.00)	
	Asian	42 (4.00)		174 (16.20)		65 (6.00)	
	Other	25 (4.00)		129 (18.30)		50 (7.00)	
	Multiple races	25 (3.00)		105 (13.70)		45 (6.00)	
**Ethnicity**			**.67**		**.11**		**.20**
	Hispanic or Latino	23 (3.00)		101 (13.00)		43 (6.00)
	Non-Hispanic	659 (3.20)		2280 (11.16)		962 (4.70)	
**Education**			**<.001**		**.04**		**.03**
	Master’s or doctoral degree	218 (2.70)		891 (11.10)		330 (4.10)
	4-year college degree or some graduate school	208 (2.90)		796 (10.90)		359 (4.90)	
	Some college or technical school	183 (3.90)		585 (12.50)		245 (5.20)	
	High school or less	78 (6.00)		149 (10.70)		82 (6.00)	
**Language**			**.10**		**.03**		**.001**
	Other	21 (5.00)		67 (15.00)		29 (6.00)	
	English	664 (3.20)		2327 (11.20)		978 (4.70)	
**General health**			**<.001**		**.001**		**<.001**
	Excellent, very good, or good	547 (3.00)		1996 (11.06)		710 (3.90)
	Fair or poor	138 (4.20)		429 (13.00)		301 (9.10)	
**Number of notes read**			**<.001**		**<.001**		**<.001**
	1	33 (2.00)		165 (10.70)		51 (3.00)
	2 or 3	239 (2.80)		904 (10.60)		369 (4.30)	
	4 or more	420 (3.80)		1331 (12.02)		614 (5.50)	
	Do not know or not sure	45 (5.00)		129 (14.50)		44 (5.00)	

^a^Proportion answering 8 to 10 on a 0-10 scale.

^b^Proportion answering *more worried*.

^c^*P* values from chi-square test of independence between categorical measures.

**Table 3:** Adjusted relative risk and 95% CI of identifying notes as very important for potential benefits.

Demographics		Taking care of health^a^ , risk ratio (95% CI)	Having an active role in care^a^, risk ratio (95% CI)	Feeling in control of care^a^, risk ratio (95% CI)	Making the most of visits^a^, risk ratio (95% CI)	Remembering the plan of care^a^, risk ratio (95% CI)	Preparing for office visits^a^, risk ratio (95% CI)
**Age (years)^b,c^**							
	18-24	1.00 (ref^d^)	1.00 (ref)	1.00 (ref)	1.00 (ref)	1.00 (ref)	1.00 (ref)
	25-44	1.14 (1.07-1.22)	1.18 (1.07-1.29)	1.04 (0.99-1.10)	1.16 (1.05-1.28)	1.05 (0.99-1.12)	1.14 (1.03-1.25)
	45-64	1.27 (1.20-1.36)	1.24 (1.13-1.36)	1.06 (1.01-1.12)	1.28 (1.16-1.40)	1.07 (1.01-1.14)	1.30 (1.18-1.42)
	65+	1.25 (1.18-1.33)	1.25 (1.14-1.38)	1.05 (0.99-1.11)	1.32 (1.2-1.46)	1.05 (0.99-1.11)	1.33 (1.21-1.45)
**Sex^b,c^**							
	Male	1.00 (ref)	1.00 (ref)	1.00 (ref)	1.00 (ref)	1.00 (ref)	1.00 (ref)
	Female	1.02 (1.00-1.04)	1.08 (1.04-1.11)	1.07 (1.05-1.09)	1.04 (1.01-1.08)	1.06 (1.04-1.08)	1.02 (1.00-1.05)
**Race^b,c^**							
	White	1.00 (ref)	1.00 (ref)	1.00 (ref)	1.00 (ref)	1.00 (rref)	1.00 (ref)
	Black	1.13 (1.09-1.18)	1.11 (1.00-1.24)	1.11 (1.07-1.16)	1.14 (1.03-1.27)	1.14 (1.08-1.19)	1.22 (1.14-1.30)
	Asian	1.11 (1.07-1.15)	1.08 (1.02-1.15)	1.07 (1.03-1.11)	1.15 (1.08-1.22)	1.12 (1.08-1.17)	1.18 (1.11-1.25)
	Multiple races	1.05 (1.01-1.10)	1.07 (1.01-1.14)	1.04 (0.99-1.08)	1.10 (1.03-1.17)	1.04 (0.99-1.09)	1.10 (1.03-1.18)
	Other	1.02 (0.98-1.07)	1.05 (0.98-1.14)	1.04 (0.99-1.09)	1.06 (0.98-1.15)	1.05 (1.00-1.10)	1.14 (1.07-1.22)
**Education^b,c^**							
	Master’s or doctoral degree	1.00 (ref)	1.00 (ref)	1.00 (ref)	1.00 (ref)	1.00 (ref)	1.00 (ref)
	4-year degree or some grad school	1.01 (0.99-1.03)	1.05 (1.01-1.09)	1.03 (1.01-1.06)	1.05 (1.01-1.09)	1.08 (1.06-1.11)	1.06 (1.02-1.09)
	Some college or technical school	1.06 (1.04-1.08)	1.09 (1.05-1.14)	1.08 (1.06-1.11)	1.09 (1.05-1.14)	1.17 (1.14-1.20)	1.23 (1.18-1.27)
	High school or less	1.11 (1.08-1.14)	1.12 (1.05-1.20)	1.15 (1.11-1.18)	1.14 (1.07-1.22)	1.23 (1.19-1.27)	1.37 (1.31-1.44)
**Ethnicity^b,c^**							
	Non-Hispanic	1.00 (ref)	1.00 (ref)	1.00 (ref)	1.00 (ref)	1.00 (ref)	1.00 (ref)
	Hispanic or Latino	1.11 (1.07-1.16)	1.02 (0.94-1.11)	1.08 (1.04-1.13)	1.02 (0.94-1.11)	1.11 (1.07-1.16)	1.14 (1.07-1.22)
**General health^b,c^**							
	Excellent or very good	1.0 (ref)	1.00 (ref)	1.00 (ref)	1.00 (ref)	1.00 (ref)	1.00 (ref)
	Good	0.95 (0.93-0.98)	0.91 (0.87-0.95)	0.91 (0.88-0.93)	0.92 (0.88-0.96)	0.99 (0.96-1.02)	0.95 (0.92-0.99)
	Fair or poor	0.98 (0.96-1.00)	0.96 (0.93-1.00)	0.95 (0.93-0.96)	0.95 (0.92-0.98)	1.01 (0.98-1.03)	0.99 (0.96-1.02)
**Primary language^b,c^**							
	English	1.00 (ref)	1.00 (ref)	1.00 (ref)	1.00 (ref)	1.00 (ref)	1.00 (ref)
	Other	1.07 (1.02-1.13)	1.09 (0.99-1.20)	1.09 (1.04-1.15)	1.12 (1.03-1.23)	1.11 (1.06-1.18)	1.28 (1.19-1.37)
**Number of notes read^b,c^**							
	1	1.00 (ref)	1.00 (ref)	1.00 (ref)	1.00 (ref)	1.00 (ref)	1.00 (ref)
	2-3	1.20 (1.14-1.26)	1.16 (1.08-1.25)	1.15 (1.09-1.20)	1.15 (1.07-1.24)	1.14 (1.09-1.20)	1.23 (1.14-1.32)
	4+	1.44 (1.37-1.51)	1.38 (1.29-1.48)	1.37 (1.31-1.43)	1.40 (1.30-1.51)	1.36 (1.30-1.43)	1.62 (1.51-1.74)
	Do not know	1.08 (1.01-1.16)	1.03 (0.92-1.15)	1.02 (0.95-1.10)	1.04 (0.93-1.17)	1.07 (0.99-1.15)	1.15 (1.03-1.28)

^a^Answering 8 to 10 on a 0-10 scale.

^b^Obtained from log Poisson regression adjusted for age, sex, race, education, ethnicity, language, self-reported health, and number of notes read.

^c^22,947 patients included in the model with no missing data on the dependent or independent variable.

^d^ref: reference group.

## Discussion

### Principal Findings

In this study, the largest assessment to date of patients who read a wide variety of clinicians’ notes over time, patients report that reading clinical notes brings them substantial benefit. The respondents represent urban and rural settings, varying education levels, and broad age and racial distributions, and they had access to notes composed by most clinicians providing primary care and ambulatory care in medical, surgical, and mental health specialties. About two-thirds of patients describe notes as extremely important in increasing their sense of control, improving recall and understanding of their plans for care, and better preparing them for visits. Few reported confusion or increased worries, and those patients from medically underserved groups reported the most benefit. Whether or not they chose to review their notes, patients overwhelmingly approved the practice, with a majority reporting that access to notes would be extremely important in determining their future choice of clinicians. On the contrary, only a third of patients recalled discussing their notes during visits or having their clinicians recommend that they read them.

### Comparison With Other Studies

These reports from almost 23,000 patients who read their notes amplify and reinforce the findings from smaller, more targeted, and shorter studies within primary care practices and discrete clinical specialties [5-7,9,12,13,21]. Many respondents had read notes over several years, and these results may foretell a *steady state* of patient experiences over the longer term. The high proportion of patients reporting benefits from reading notes did not diminish compared with our first survey in primary care patients several years ago, and more patients reported mentioning their note reading to their providers (32%, up from 19%) [3].

### Implications of the Findings

Focusing on clinically important process measures, these results strongly suggest that transparency helps patients feel more engaged in their care. This is an important finding, given that a growing body of evidence indicates that engaged patients are more likely to adhere to treatment plans and medications, follow through on screening and prevention protocols, detect and prevent errors, and adopt more effective management strategies for chronic illnesses [22-26].

Although the time-honored principle of patient-clinician confidentiality is not in itself affected by open notes, it is up to patients to decide whether or not to disclose their medical information to others. More than a third of respondents reported sharing notes with someone else, almost twice the rate reported in 2012 in the study of primary care patients in the same institutions [3]. Shared notes may be particularly helpful to informal care partners [27,28] and to those in search of informal second opinions from both lay and professional associates.

In this survey, patients who are potentially the most vulnerable—those who are older, less educated, non-white, Hispanic, or not English speakers at home—reported the most benefit. By virtue, both of taking the initiative to sign onto a patient portal and reading their notes, they may not be representative of other Americans with similar demographics, as is true for everyone in our sample. But this finding extends and amplifies findings in earlier inquiries [5-7,9,10,12,13,21]. Vulnerable patients may come to a visit with a lower baseline sense of control and knowledge than others; they may also have more difficulty understanding or retaining what practitioners say or emphasize. Assisted at times by family members or other acquaintances who can help interpret and research points made in notes, the possibility of review in their homes may contribute to their particular enthusiasm for this new opportunity. Moreover, in several studies, patients who are disadvantaged may trust their health professionals less than those with social and educational backgrounds similar to their clinicians [29]. Shared notes can increase trust, as studies and anecdotes from both patients and clinicians suggest [24,26]. That particularly vulnerable patients may gain the most from open notes is worthy of further inquiry. Similarly, the fact that minority patients are more concerned about privacy than white patients also deserves further study.

As some of our findings suggest, embedding open notes into clinical practice faces many challenges. Even in these 3 *mature* institutions, the majority of their patients have not registered on their patient portals, and half of the responding nonreaders did not know that notes were available—even though they were using their portals for other purposes. Moreover, contrary to our predictions, interchange in the clinician’s office about past notes was infrequent. Here, there were also substantial differences across the sites. Patients from rural Pennsylvania were less likely than those in Boston and Seattle to report speaking with their providers about note reading. However, they were also more likely to report benefits from reading, including greater feelings of control of their care and better preparation for office visits. Access to clinicians’ notes may offer particular benefits to rural populations, a possibility that warrants further study.

### Strengths and Limitations

This is a cross-sectional study examining patients’ self-reported experiences from only 3 regions of the United States, and results may not be generalizable to other regions or practices. Moreover, the response rate was modest, although it was similar to the response rate in a recent Consumer Assessment of Healthcare Provider and Systems survey [30]. It is possible that the survey respondents were those most enthusiastic about open notes. Although there were some demographic differences between responders and nonresponders, these differences were small, and the size of the sample analyzed gives further weight to the findings. It should be noted also that the majority of respondents were white, in good health, and highly educated. It is difficult to draw firm conclusions about the benefits to non-English speakers because the survey was only offered in English; subsequent surveys could be administered in other languages and also explore issues of health literacy. Finally, the literature on the impact of portals on patients is often confused by lack of specificity about different functionalities. A strength of our study is that we took several steps to make sure patients had experience reading notes and were reporting on reading visit notes, rather than on other information available on their portals.

### Unanswered Questions and Future Directions

Some argue today that fully open medical records are simply *...the right thing to do*, and this large survey of patients furnishes further evidence that their benefits outweigh their risks, certainly from the point of view of patients. Although the findings confirm that the benefits of note reading extend beyond primary care practices to virtually all specialties and types of clinicians, more needs to be learned about using open notes as a tool for communication and promoting interaction between patients and clinicians across health care venues and populations. The Department of Health and Human Services has proposed new rules that would increase patient access and control of their medical information, and easy electronic access to notes could become the law of the land [31]. To what degree can active educational interventions help patients learn optimally from what their records document? And in the future, might patients also contribute to their records by providing interval histories and articulating their goals for a visit in their own words, thereby enriching narratives, promoting focused interactions, and hopefully off-loading work from beleaguered clinicians? [24].

At a time when medical practice in the United States is moving toward shared notes as a new standard, the patients participating in this study offer both affirmative and provocative reports, but building shared notes into the fabric of care remains a work in progress.

## Abbreviations

BIDMC: Beth Israel Deaconess Medical Center
PCP: primary care physician
UW: University of Washington Medicine
