# Correction: OpenNotes After 7 Years: Patient Experiences With Ongoing Access to Their Clinicians’ Outpatient Visit Notes

**DOI:** 10.2196/18639

**Published:** 2020-04-30

**Authors:** Jan Walker, Suzanne Leveille, Sigall Bell, Hannah Chimowitz, Zhiyong Dong, Joann G Elmore, Leonor Fernandez, Alan Fossa, Macda Gerard, Patricia Fitzgerald, Kendall Harcourt, Sara Jackson, Thomas H Payne, Jocelyn Perez, Hannah Shucard, Rebecca Stametz, Catherine DesRoches, Tom Delbanco

**Affiliations:** 1 Division of General Medicine Beth Israel Deaconess Medical Center Boston, MA United States; 2 Harvard Medical School Boston, MA United States; 3 College of Nursing and Health Sciences University of Massachusetts Boston, MA United States; 4 David Geffen School of Medicine University of California Los Angeles Los Angeles, CA United States; 5 Division of General Internal Medicine School of Medicine University of Washington Seattle, WA United States; 6 Department of Medicine Medicine Information Technology Services University of Washington Seattle, WA United States; 7 Steele Institute for Health Innovation Geisinger Danville, PA United States

In “OpenNotes After 7 Years: Patient Experiences With Ongoing Access to Their Clinicians’ Outpatient Visit Notes: (J Med Internet Res 2019;21(5):e13876), there were errors which were not identified during the proofing stage.

The original published Methods did not include the following sentence about the excluded respondents:

We also excluded respondents who reported reading notes for a week or less, or did not answer the question about length of time reading notes, since our objective was to assess patients’ experiences over the prior 12 months.

The new sentence has now been added to Methods of the paper under the subheading “Statistical Analysis”, as follows:

Statistical Analysis

To maximize the chances that we were including responses about clinical notes rather than another part of the record, as a final step, we excluded responses from patients whose self-report of note reading in the past 12 months did not match portal data; for example, patients reported they had read notes, but the portal tracking data showed they had not. We also excluded respondents who reported reading notes for a week or less, or did not answer the question about length of time reading notes, since our objective was to assess patients’ experiences over the prior 12 months.

Additionally, the original published Results incorrectly included the following sentence under the subheading “Accessing and Reading Notes”:

After excluding those with unconfirmed note reading status, 23,710 responses were included in the analysis: 22,947 note readers and 763 nonreaders. Among note readers, three-quarters reported reading notes for a year or more and half reported reading 4 or more notes.

This sentence has been corrected to the following:

After note reading exclusions, 23,710 responses were included in the analysis: 22,947 note readers and 763 nonreaders. Among note readers, three-quarters reported reading notes for a year or more and half reported reading 4 or more notes.

Finally, in the flowchart in Figure 1, the bottom left box included the following incorrect wording:

5,072 Discordant information about note reading status

4,443 Portal data conflicts with self-report of reading

629 Do not remember reading notes in the past 12 months, or missing

This wording has been corrected to the following:

5,072 Note reading exclusions

4,443 Portal data conflicts with self-report of reading

629 Reading ≤1 week or missing

The corrected Figure 1 can be seen below.

**Figure 1:**
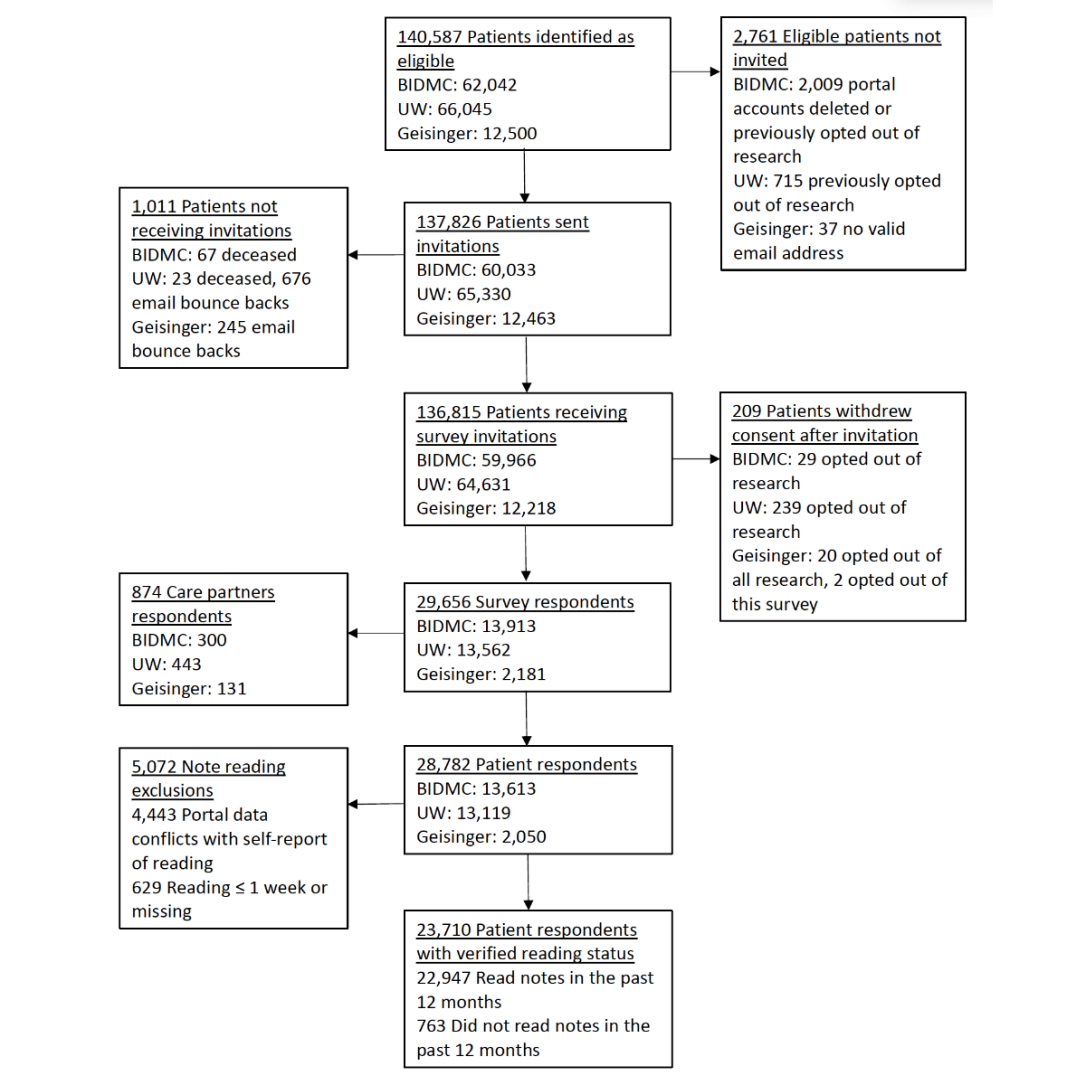
Study flow diagram. BIDMC: Beth Israel Deaconess Medical Center; UW: University of Washington Medicine.

The correction will appear in the online version of the paper on the JMIR website on April 30, 2020, together with the publication of this correction notice. Because this was made after submission to PubMed, PubMed Central, and other full-text repositories, the corrected article has also been resubmitted to those repositories.

